# Using Weibo and WeChat social media channels to assess public awareness and practices related to antimicrobial resistance, China, 2019

**DOI:** 10.1186/s12889-021-10648-5

**Published:** 2021-05-14

**Authors:** Lei Wang, Sujian Situ, Jeanette J. Rainey, Bin He, Xiaoge Su, Ronald L. Moolenaar, Ying Cui

**Affiliations:** 1 Health Communication Center (National 12320 Health Hotline Management Office), Chinese Center for Disease Control and Prevention, No.155 Changbai Rd, Changping District, Beijing, China; 2 Division of Global Health Protection, Center for Global Health, United States Centers for Disease Control and Prevention, Beijing, China; 3 Division of Global Health Protection, Center for Global Health, United States Centers for Disease Control and Prevention, Atlanta, GA USA

**Keywords:** AMR, Antibiotic resistance, Online survey, China

## Abstract

**Background:**

Antimicrobial resistance (AMR) is a global healthcare problem, including in China where high rates of resistance to common bacterial infections have been documented. In 2016, the National Health and Family Planning Commission (NHFPC) in China established a comprehensive strategic plan to increase awareness about AMR through education programs.

**Methods:**

We conducted an online survey to assess changes in public knowledge, awareness and practices related to AMR in China since 2016. The survey was administered using China’s national and provincial level 12320 Health Hotline Weibo (micro-blog site) and WeChat (text messaging service) social media accounts from April 12, 2019 to May 7, 2019. All persons ≥16 years of age able to read Chinese were eligible to participate.

**Results:**

A total of 2773 respondents completed the survey. Of the 2633 respondents indicating recent use of antibiotics, 84% (2223) reported obtaining their course of antibiotics from a hospital or pharmacy, 9% (246) of respondents reported using antibiotics saved from a previous prescription or treatment course, and 42% (1115) of respondents reported that they had stopped taking antibiotics as soon as they started feeling better. Most respondents correctly indicated that antibiotics can effectively treat urinary tract infections (86% [2388]) and skin infections (76% [2119]), but many incorrectly indicated that antibiotics can also treat viral infections such as measles (32% [889]) or a cold or flu (26% [726]). Of all respondents, 95% (2634) had heard of ‘antibiotic resistance’. Almost half (47% [1315]) reported using antibiotics within the last 6 months.

**Conclusion:**

While awareness of AMR was high in this survey of social media users in China, inappropriate antibiotic use remains common, including the believe that antibiotics can effectively treat viral infections. Multiple interventions targeting the correct use of antibiotics and information on the cause AMR are likely needed. The 12320 Health Hotline provides a platform for conducting routine surveys to monitor antibiotic use and knowledge about AMR.

**Supplementary Information:**

The online version contains supplementary material available at 10.1186/s12889-021-10648-5.

## Background

Antimicrobial resistance (AMR) is the acquisition of mutations in microorganisms that renders treatment with first-line antibacterial, antiviral, antiparasitic, and antifungal drugs ineffective [1]. Development of AMR is associated with the improper use of antibiotics, including overprescribing and incomplete treatment [1], leading to increases in morbidity and mortality, as well as increased medical costs [1, 2]. Antimicrobial resistance contributed to more than 700,000 deaths globally in 2013 [1], and many common infections have already developed resistance to one or more first line antibiotic treatments [2]. In China, antimicrobial resistance is a public health priority [3], where almost 8% of all multi-drug resistant TB infections are extensively resistant (XDR) [4] and an increasing percentage of health-care-associated infections are caused by resistant pathogens, such as methicillin-resistant *Staphylococcus aureus* (MRSA) [3, 5]. Several studies, including from China, have shown *Escherichia coli* (*E. coli*) resistance to 3rd generation cephalosporins as well as colistin, the last-line antibiotic for treating gram-negative infections [2, 6, 7]. The unnecessary or excess use of antibiotics in health care in China has been documented [8–11].

The National Health and Family Planning Commission (NHFPC) in China developed a comprehensive AMR National Action Plan in 2016 [12]. The plan included steps to increase awareness about AMR through traditional education programs as well as through new social media and web-based platforms. Various national and provincial level offices have used these approaches to publicly disseminate material on appropriate antibiotic use. The National 12320 Health Hotline management office was established as part of the Chinese Center for Disease Control and Prevention (China CDC) in 2005 [13]. The Health Hotline management office maintains Weibo and WeChat social media accounts and supports call-centers in provinces and autonomous regions in mainland China. Weibo is a microblogging service in China that allows users to post and share short messages, and attach links, images, or videos to the messages [14]. The official Weibo site of the National 12320 Health Hotline management office had more than 6.3 million followers in 2018. The Health Hotline management office also maintains an official WeChat account. Individuals and organizations can use WeChat as a text messaging, photo sharing, and mobile payment app (https://www.wechat.com).

Hotline staff leverages this online presence to provide information on important public health topics, including AMR. In this project, therefore, we conducted a low-cost and reproducible online survey using these social media platforms with the aim of assessing community knowledge, awareness and practices related to antibiotic use. Routine surveys such as this are essential for describing trends and evaluating the impact of interventions on priority public health topics. Findings from the survey can help public health officials improve the design and implementation of AMR education campaigns.

## Methods

### Target population

We developed and administered a national online survey using the 12320 Health Hotline’s Weibo and WeChat accounts and the Health Hotline’s official website. All persons accessing these sites during the project period from April 12–May 7, 2019, could directly access a link to the survey. Participation was completely voluntary. Participants were required to be at least 16 years of age, review the instructions in Chinese, and provide consent before initiating the survey. Participants were asked to participate only once, but because we were unable to crosscheck participants on the two different social media platforms (Weibo and WeChat), participants could have feasibly responded more than once. Participants indicated consent by checking the eligibility and agreement requirements on the landing page of the online survey. Persons not meeting these requirements (e.g., under 16 years of age) were unable to complete the survey. As described in the survey instructions, we randomly selected 100 respondents at the end of the project period to receive a small gift (value of US$5–6). This post-survey gift was offered as an incentive to increase the response rate [15, 16].

The initial goal for survey completion, based on the methodology outlined in the 2015 World Health Organization (WHO) survey report [11], was set at > 1000 respondents. As described in the report, a sample size of 1000 respondents would generate robust data, while also ensuring resource effectiveness. The project period (April 12 – May 7) was the amount of time likely needed to reach a minimum of 1000 respondents. Project staff monitored survey participation on a weekly basis. To increase participation among persons in western and central China, which are likely to be more rural, the National Health Hotline management office provided a link to the survey on the provincial level 12320 Health Hotline Weibo and WeChat accounts in these regions. Our approach resulted in a convenience sample of persons visiting the Health Hotline’s Webio and WeChat social media sites during the project period. Although we were unable to generate statistical comparisons between subsets of the survey population, this sampling approach would allow us to identify and compare general patterns of antibiotic use and AMR awareness to previous surveys in China and elsewhere.

### Online survey

We selected ten questions (Additional file 1) from the 2015 WHO survey [11] and divided these questions into the following four sections for the China AMR survey: (1) demographic information, (2) previous practices related to antibiotic use, (3) knowledge about antibiotics, and (4) awareness about AMR. Only multiple choice or true and false questions were included in the survey to minimize participant burden. All completed response data were saved on the National 12320 Health Hotline Office’s website, Weibo and WeChat accounts.

### Data management and analysis

At the end of the three-week period, we downloaded and combined participant data from the Health Hotline’s website, Weibo, and WeChat accounts for cleaning and analysis. We calculated responses for each question and compared these responses by age (16–24, 25–44, 45–64, 65+ years), sex (male or female), place of residence (urban, suburban, rural), education level (less than 12th grade, high school graduate, some college or associate degree, bachelor’s degree, or master’s, professional, or doctoral degree), and region (Eastern, Central, or Western according to the respondents’ province) (Additional file 2). We aggregated the number of respondents that either strongly or slightly agreed (among the following choices: agreed strongly, agreed slightly, neither agreed or disagreed, disagreed slightly, or disagreed strongly) in our analysis about statements on antibiotic resistance and approaches for prevention. We present the results for each of the four sections of the survey as outlined above. Because participants represented a convenience rather than a probability sample, we only present results as the number of respondents and percentages of the total or a specific subset of the surveyed population.

As an evaluation activity, the survey was exempt from review by the Ethical Review Board at China CDC. The project was reviewed in accordance with the United States Centers for Disease Control and Prevention (US CDC) human research protection procedures and was determined to not constitute human-subjects research by the US CDC Center for Global Health. All personal identifying information obtained from the online survey was maintained in a secure location and removed from the project database prior to analysis and preparation of all reports and manuscripts.

## Results

From April 12–May 7, 2019, we recorded 4983 clicks on the survey link. Of these clicks, 56% (2773) resulted in a completed AMR survey. Of the responders completing the survey, the majority were female (64% [1782]), between 25 and 44 years of age (71% [1963]), and from the Eastern Region of China (79% [2177]). This survey population was more female, middle-aged, urban, and from the Eastern Region than the general population in China (Table 1).

**Table 1:** Demographic characteristics of respondents to an online survey about antibiotic resistance administered by the National 12320 Health Hotline management office, China, April 12–May 7, 2019

	Survey Respondents	China Population^**a**^
**Characteristics**	**N (%)**	**N (%)**
**Sex**		
**Male**	991 (36)	711(51)
**Female**	1782 (64)	679 (49)
**Age (years)**		
**16–24**	156 (6)	227 (20)
**25–44**	1963 (71)	441 (40)
**45–64**	586 (21)	324 (29)
**≥65**	68 (3)	119 (11)
**Provinces**		
**Eastern**	2177 (79)	577 (42)
**Central**	368 (13)	434 (31)
**Western**	228 (8)	377 (27)
**Place of Residence**		
**Urban**	2098 (76)	404 (30)
**Sub-urban**	507 (18)	266 (20)
**Rural**	168 (6)	663 (50)
**Education**^**b**^		
**Less than High School**	167 (6)	–
**High School Graduate**	354 (13)	–
**Some Junior College**	694 (25)	–
**Some Undergraduate**	1277 (46)	–
**Some Graduate**	281 (10)	–
**Total**	2773 (100)	–

^a^In millions

^b^National data not available for education level

### Practices related to antibiotic use

Of the total 2773 respondents, 20% (541) of respondents had taken antibiotics within the last month, 28% (774) within the previous two to 6 months, and an additional 13% (364) within the previous seven to 12 months (Table 2). Although more than half of all respondents reported obtaining their most recent course of antibiotics from a hospital or pharmacy (Table 3), 9% (246) of respondents reported using antibiotics saved from a previous prescription or treatment course. This finding was primarily driven by urban respondents and those with a higher level of education (i.e., a lower percent of rural and less educated respondents reported savings antibiotics from a previous prescription or treatment course compared to other respondents). The majority (62% [1634]) of respondents indicated that they received advice on the use of antibiotics from a medical professional or pharmacist. At the same time, 42% (1115) of respondents reported that they would stop taking antibiotics as soon as they started feeling better. This finding was inversely related to a respondent’s education level. That is, a larger percent of respondents with more than some junior college reported stopping the treatment as soon as they felt better compared to those with a lower education level.

**Table 2:** Timing of most recent antibiotic use as reported by respondents to an online survey on antibiotic resistance administered by the National 12320 Health Hotline management office, China, April 12–May 7, 2019

Characteristics	Respondents N	In last month N (%)	Within last 2–6 months N (%)	Within last 7–12 months N (%)	> 1 year ago N (%)	Never N (%)	Do not recall N (%)
**Sex**							
**Male**	991	188 (19)	275 (28)	140 (14)	252 (25)	69 (7)	67 (7)
**Female**	1782	353 (20)	499 (28)	224 (13)	523 (29)	71 (4)	112 (6)
**Age**							
**16–24**	156	32 (22)	34 (22)	10 (6)	37 (24)	22 (14)	21 (14)
**25–44**	1963	380 (19)	570 (29)	257 (13)	559 (29)	85 (4)	112 (6)
**45–64**	586	109 (19)	153 (26)	91 (16)	162 (28)	33 (6)	38 (7)
≥**65**	68	20 (29)	17 (25)	6 (9)	17 (25)	0 (0)	8 (12)
**Provinces**							
**Eastern**	2177	410 (19)	598 (28)	303 (14)	632 (29)	99 (5)	135 (6)
**Central**	368	84 (23)	102 (28)	39 (11)	90 (25)	31 (8)	22 (6)
**Western**	228	47 (21)	74 (33)	22 (10)	53 (23)	10 (4)	22 (10)
**Place of residence**							
**Urban**	2098	409 (20)	601 (29)	275 (13)	595 (28)	101 (5)	117 (6)
**Suburban**	507	98 (19)	135 (27)	69 (14)	141 (28)	20 (4)	44 (9)
**Rural**	168	34 (20)	38 (23)	20 (12)	39 (23)	19 (11)	18 (11)
**Education**							
**Less than High School**	167	37 (22)	38 (23)	21 (13)	28 (17)	22 (13)	21 (13)
**High School Graduate**	354	60 (17)	96 (27)	48 (14)	94 (27)	31 (9)	25 (7)
**Some Junior College**	694	140 (20)	211 (30)	86 (13)	181 (26)	29 (4)	47 (9)
**Some Undergraduate**	1277	257 (20)	356 (28)	170 (13)	381 (30)	44 (3)	69 (5)
**Some Graduate**	281	47 (16)	73 (26)	39 (14)	91 (32)	14 (5)	17 (6)
**Total**	2773	541 (20)	774 (28)	364 (13)	775 (28)	140 (5)	179 (7)

**Table 3:** Source and use of antibiotics by respondents to an online survey on antibiotic resistance administered by the National 12320 Health Hotline management office, China, April 12 to May 7, 2019. Excludes respondents who reported never taking antibiotics (*n* = 140)

Characteristics	Where did respondent obtain antibiotics when last used?						When did respondent stop taking antibiotics?		
	Hospital N (%)	Pharmacies N (%)	Saved from before N (%)	Family or Friend N (%)	Internet N (%)	Do not recall N (%)	As Directed N (%)	Feeling Better N (%)	Do Not Recall N (%)
**Sex**									
**Male**	498 (54)	289 (31)	78 (9)	19 (2)	4 (< 1)	34 (4)	497 (54)	396 (43)	29 (3)
**Female**	992 (58)	444 (26)	168 (10)	26 (2)	3 (< 1)	78 (5)	956 (56)	719 (42)	36 (2)
**Age**									
**16–24**	68 (56)	43 (32)	5 (4)	5 (4)	1 (< 1)	12 (9)	74 (55)	56 (42)	4 (3)
**25–44**	1049 (58)	551 (30)	175 (10)	26 (1)	5 (< 1)	72 (4)	1030 (55)	798 (42)	50 (3)
**45–64**	322 (61)	130 (24)	61 (11)	14 (3)	1 (< 1)	25 (5)	310 (56)	233 (42)	10 (2)
≥**65**	51 (79)	9 (13)	5 (7)	0 (0)	0 (0)	3 (4)	39 (57)	28 (41)	1 (2)
**Provinces**									
**Eastern**	1247 (60)	507 (24)	202 (10)	36 (2)	5 (< 1)	81 (4)	1147 (55)	881 (42)	50 (3)
**Central**	144 (43)	141 (42)	27 (8)	5 (2)	1 (< 1)	19 (6)	188 (56)	139 (41)	10 (3)
**Western**	99 (45)	85 (39)	17 (8)	4 (2)	1 (< 1)	12 (6)	118 (54)	95 (44)	5 (2)
**Place of Residence**									
**Urban**	1133 (57)	549 (28)	199 (10)	33 (2)	7 (< 1)	76 (4)	1114 (56)	841 (42)	42 (2)
**Sub-urban**	277 (57)	138 (28)	38 (8)	8 (2)	0 (0)	26 (5)	262 (54)	207 (42)	18 (4)
**Rural**	80 (54)	46 (31)	9 (6)	4 (3)	0 (0)	10 (7)	77 (52)	67 (45)	5 (3)
**Education**									
**Less than High School**	77 (53)	50 (35)	5 (3)	3 (2)	0 (0)	10 (7)	64 (44)	72 (50)	9 (6)
**High School Graduate**	175 (54)	98 (30)	23 (7)	7 (2)	0 (0)	20 (6)	168 (52)	145 (45)	10 (3)
**Some Junior College**	336 (51)	203 (31)	78 (12)	12 (2)	3 (< 1)	33 (5)	342 (51)	307 (46)	16 (2)
**Some Undergraduate**	746 (61)	321 (26)	106 (9)	18 (2)	3 (< 1)	39 (3)	708 (57)	500 (41)	25 (2)
**Some Graduate**	156 (58)	61 (23)	34 (13)	5 (2)	1 (< 1)	10 (4)	171 (64)	91 (34)	5 (2)
**Total**	1490 (57)	733 (28)	246 (9)	45 (2)	7 (< 1)	112 (4)	1453 (55)	1115 (42)	65 (2)

### Knowledge about antibiotics

**Fig. 1:**
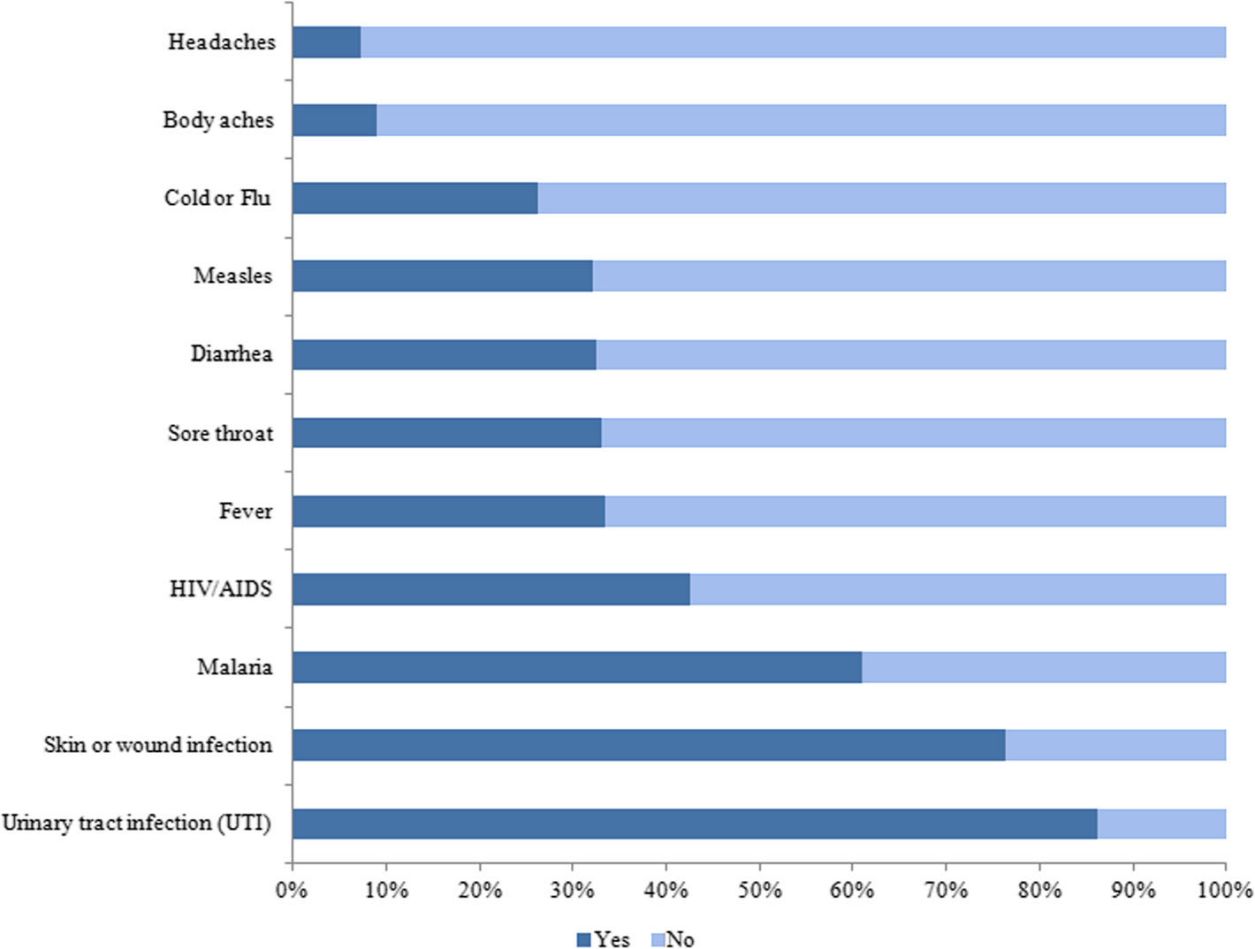
Conditions that could be treated by antibiotics according to respondents to an online survey antibiotic resistance, administered by the National 12320 Health Hotline management office, China, April 12 to May 7, 2019 (*N* = 2773)

Most respondents correctly indicated that antibiotics could treat urinary tract infections (86% [2388]) and skin infections (76% [2119]). Of all 2773 respondents, more than a quarter of respondents also incorrectly indicated that antibiotics could effectively treat viral infections such as measles (32% [889]), or a cold or influenza (26% [726]) (Fig. 1). Almost 50% of younger respondents 16 to 24 years of age and 38% (combined data not shown) of those with a high school education or less indicated that antibiotics could effectively treat measles. Similar results on perceived usefulness of antibiotics for treating a cold of influenza were observed for younger respondents and respondents with a lower level of education.

### Awareness about AMR

Of all 2773 respondents, 95% (2634) had heard of ‘antibiotic resistance’. Respondents less than 25 years of age (90% [140/156]), those with less than a high school education (86% [143/167]) and those residing in rural areas (86% [145/168]) were slightly less likely to have heard of the term compared to other groups. Of the 2634 respondents who had heard of AMR, most (76% [2009]) had heard about antibiotic resistance from television, radio and social media. Other important sources included healthcare staff (45% [1198]) and family and friends (29% [770]).

**Fig. 2:**
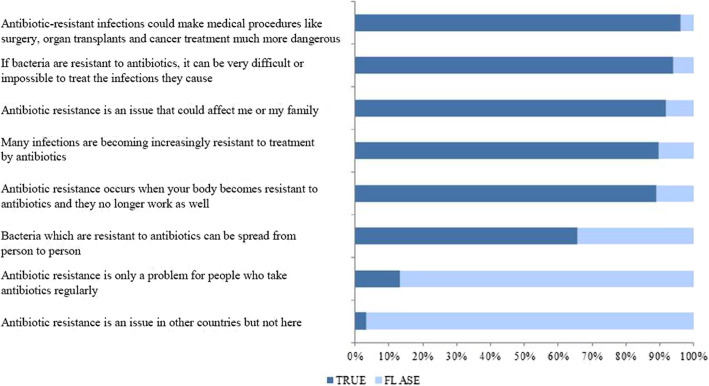
Cause of antibiotic resistance according to respondents to an online survey on antibiotic resistance, administered by the National 12320 Health Hotline Management Office, China, April 12 to May 7, 2019 (*N* = 2773)

**Fig. 3:**
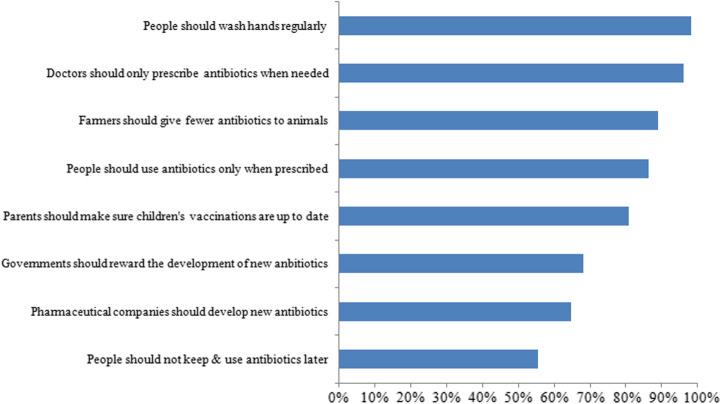
Actions believed to help address the problem of antibiotic resistance according to respondents to an online survey on antibiotic resistance, administered by the National 12320 Health Hotline Management Office, China, April 12–May 7, 2019 (*N* = 2773)

Respondents’ awareness and knowledge about AMR are presented in Fig. 2. Of all respondents, 89% (2459) incorrectly thought that AMR occurred when “your body becomes resistant to antibiotics and they no longer work well”. At the same time, 65% (1814) of respondents correctly indicated that “bacteria that are resistant to antibiotics can spread from person to person”. Almost all respondents (98% [2723]) reported that people could help slow the development of AMR by good hand hygiene practices (e.g., preventing transmission of infections that could lead to antibiotic use), and that doctors should only prescribe antibiotics when needed (96% [2662]) (Fig. 3). More than half (55% [1539]) of respondents agreed that people should avoid saving antibiotics from one illness for later use (Additional file 3).

## Discussion

Antibiotic stewardship is defined as effectively coordinating strategies aimed at appropriate use of antibiotic medications, reducing resistance to available antibiotics, and decreasing unnecessary health care costs associated with treating resistant infections [1, 2]. In 2016, the Chinese government developed a comprehensive plan to address AMR [12, 17] that outlined steps for increasing public awareness and knowledge about the appropriate use of antibiotics. Our online survey among Chinese social medial users suggests that additional work is needed in increasing knowledge, awareness, and appropriate practices about the use of antibiotics. While the majority of respondents had heard of AMR, many reported improper antibiotic use. Differences in responses between urban and rural settings, as well as by educational level, suggest that different intervention approaches should be developed to target specific segments of society. This conclusion is consistent with findings from previous community surveys on AMR in China and elsewhere [18–21]. Routine online surveys such as ours provide a low-cost and reproducible approach to monitor and evaluate changes in antibiotic use and public knowledge and awareness over time.

In our survey, almost half (47%) of respondents reported using antibiotics within the last 6 months, compared to 57% of Chinese participants who reported using antibiotics during the same time period in the 2015 WHO survey; both country-specific estimates are lower than the overall mean of 65% calculated from the 12 countries participating in the WHO survey [11]. Unnecessary antibiotic use has been observed in previous surveys conducted in China, particularly among university students, who reported frequent prescription and non-prescription use of antibiotics [19, 21, 22]. This includes one study in which 64% of surveyed medical students maintained their own stock on antibiotics and 54% self-medicated with antibiotics for a mild or potentially self-limiting illness [22]. Frequent antibiotic use may also be driven by pressure on clinicians to provide a treatment, even if the infection is not suspected to be of bacterial etiology [9], and patients specifically ask for an antibiotic. We found that nearly one third of respondents believed antibiotics are effective treatments for the common cold and influenza viruses. Strategies to support clinicians who adhere to existing antibiotic prescription guidelines could help reduce the use of antibiotics when not medically warranted [23].

Since 2016, China has implemented AMR training programs [24] and education campaigns, including live web-chat sessions between experts and the general community through the Weibo Hotline. Although our results suggested improved knowledge about the appropriate use of antibiotics compared to the previous WHO survey, a recent survey of pharmacy customers in three urban centers in China reported that respondents continued to have a poor understanding about antibiotics [25]. These differences in knowledge about antibiotics could reflect variability in age and education level among survey respondents. In our survey, for example, a larger percent of respondents 16 to 24 years of age (49%) and of those with a high school education or less (38%) reported that antibiotics could be used to treat viral infections compared to the other demographic groups. National surveys on AMR in Japan [18] and Thailand [26] reported similar findings. Developing and disseminating education material on the role of antibiotics targeting these specific age- and education-level groups is likely to help address these knowledge gaps.

Obtaining antibiotics and advice from hospital or pharmacy staff was common among respondents; however, almost 11% of respondents reported obtaining antibiotics from family or friends or that they used antibiotics saved from a previous prescription. This is higher than global estimates from the WHO survey, where approximately 5% of all respondents obtained antibiotics from these non-health care related sources [11]. Interestingly, highly educated respondents (those with at least some junior college work), and those residing in urban areas, were more likely to use antibiotics saved from a previous treatment course. Additionally, more than 40% of respondents, including almost one third of respondents with some graduate schoolwork, indicated that they had stopped taking the antibiotic treatment as soon as they started feeling better. Similar findings were reported in previous surveys among college students in China [19, 21, 22]. Several factors, including a higher perception of self-reliance, time constraints, as well as costs associated with hospital visits and long waiting times, may contribute to this finding [19, 21, 22]. Improving patient adherence to clinical recommendations will be useful, but a multi-faceted approach that also includes additional training of health care and pharmacy staff on antibiotic prescription guidelines, reducing availability and use of non-prescription antibiotics, and increasing public awareness on the differences between bacterial and viral infections will likely also be required [27]. Identifying alternative access to health care services for less serious ailments (e.g., live online chats or telephone contact with medical staff) could help discourage unnecessary self-treatment with antibiotics.

Most respondents (95%) to our online survey were familiar with the term antibiotic resistance. These Chinese survey respondents were more familiar than those in Japan (41%) [18] and in Thailand (18%) [26] as well as those participating in the 2015 WHO survey (range: 22–89%) [11]. Interestingly, respondents in China were most likely to hear about AMR from television, radio and social media sources. Less than half of respondents had heard about AMR from health care staff, suggesting that more work is needed to engage health care organizations in educating the public about antibiotic resistance. This is particularly important since many respondents to our survey, including those with a lower education level or residing in a rural area, incorrectly believed that antibiotic resistance occurs when their body becomes resistant to antibiotics rather than when the bacteria become resistant. As observed in previous surveys [11, 18], a majority of these same respondents also reported that good hand hygiene and vaccinations could help prevent AMR, doctors should only prescribe antibiotics when needed, and antibiotics should not be saved for use during future illnesses. Although awareness about AMR was high among urban and educated respondents in our survey, these same respondents were also the most likely to save antibiotics from a previous treatment course and stop taking antibiotics once they started feeling better. This finding further highlights the need for a multi-faceted approach targeting the supply, access, as well as demand for antibiotics; that is, increasing awareness about AMR alone may not be enough for behavioral change [27].

Findings from this survey can help inform efforts to increase public awareness and knowledge about AMR and ultimately reduce demand and inappropriate use of antibiotics. Although evidence from evaluations of previous AMR awareness campaigns suggests that these efforts are typically associated with improved awareness [28], the overall impact on decreasing antibiotic use has been limited [29–31]. In certain situations, such campaigns increased consumption due to greater information on availability of antibiotics [31] or due to fear created by the educational messages and perceived need to protect one’s self [23]. This is similar to our survey, which found that most respondents had heard of AMR from television, radio, or social media. Nevertheless, our findings suggest that future campaigns in China should focus on three key messages: 1) antibiotics do not treat viral infections such as the common cold, influenza, or measles; 2) antibiotic resistance is related to the resistance of bacteria to certain drugs and not resistance of the person taking the antibiotics; and 3) patients should complete the full course of antibiotics as prescribed by the clinician or pharmacist. Our findings, in addition to previous surveys [18–21], also suggest that these educational messages should be adapted for different segments of the population – particularly for various urban-rural settings and educational attainment levels. The National 12320 Health Hotline Management Office can use existing online and social media channels to support the development and pilot testing of these targeted messages before wider dissemination [29–31]. The Health Hotline Management Office can repeat this low-cost online survey in the future. Findings from such routine surveys can help monitor the use of antibiotics and evaluate the effectiveness of interventions on changes in antibiotic use and AMR awareness over time.

As with most online surveys, this project involved a few limitations. First, the survey population was a convenience sample and not representative of the general population in China. We attempted to recruit participants from all age groups and from both rural and urban settings by promoting the survey on provincial level Health Hotline Weibo and WeChat accounts, including in Western China and rural parts of the country (where the population is likely to be older). However, the final survey population was more middle-aged and more urban than the general population in China. Complementary telephone or house-to-house surveys may be needed to increase participation among the rural and older populations who are typically less likely to use social media. Despite this limitation, visitors to the Health Hotline Weibo and WeChat accounts could serve as a sentinel population for monitoring trends in antibiotic use and AMR awareness. This population can be reached through low-cost and reproducible routine online surveys through these existing platforms. Additionally, the survey was completed by persons visiting the Health Hotline Weibo and WeChat accounts and such respondents might already have a good awareness of public health issues. As a result, our findings might reflect a higher level of awareness about AMR than the general population in China. The survey used closed-ended questions (e.g., multiple choice, true or false) to reduce participant burden and increase participation. This approach limited the ability to fully explore participants’ understanding and perceptions of AMR. Qualitative research using open-ended questions might add a deeper understanding of methods to reach the population with appropriate health messages. Nevertheless, our results provide insight into knowledge of antibiotic use and AMR among users of social media in China. This insight can be used to develop more effective interventions in the future [24, 27].

## Conclusion

We conducted an online survey using social media platforms to assess public knowledge, awareness and practices related to antibiotic use and AMR in China. Our survey population was recruited from the Health Hotline’s Weibo and WeChat accounts and were likely more informed about health issues than the general population. Despite this limitation, our findings suggest that additional intervention programs are needed to decrease the unnecessary use of antibiotics in China. Development and piloting of interventions targeting specific segments of the population (e.g., older and rural populations) could help decrease inappropriate use of antibiotics in China. These interventions programs should incorporate three key messages: 1) antibiotics do not treat viral infections such as the common cold, influenza, or measles; 2) AMR is related to the resistance of bacteria to certain drugs and not the person taking the antibiotics; and 3) patients should complete a course of antibiotics as prescribed by the clinician or pharmacist. The 12320 Health Hotline provides a platform for conducting routine surveys to monitor and evaluate the effectiveness of these interventions.

## Supplementary Information

**Additional file 1.** Survey questions used to assess public awareness and practices related to antimicrobial resistance, China, 2019 (English version of survey questions).

**Additional file 2.** Provincial Map of China, by Region, 2019 (Map highlighting location of provinces within each of the three main regions in China – Eastern, Central, and Western China).

**Additional file 3.** Respondent feedback on approaches to help address antibiotic resistance from an online survey on antibiotic resistance, China, April 12–May 7, 2019 (Tables a, b).

## Data Availability

De-identified data collected from this online survey are available following approval from the China CDC. Interested researchers can submit a formal request and proposed use of the data by contacting the first author, Wang Lei (angelyyl@163.com), at the 12320 Health Hotline Management Office.

## Abbreviations

AMR: Antimicrobial resistance
China CDC: Chinese Center for Disease Control and Prevention
MRSA: methicillin-resistant *Staphylococcus aureus*
US CDC: United States Centers for Disease Control and Prevention
WHO: World Health Organization
XDRTB: Extensively drug-resistant tuberculosis
