# Supplementary material for: Using Weibo and WeChat social media channels to assess public awareness and practices related to antimicrobial resistance, China, 2019
Source: BMC Public Health. 2021 May 14;21:921. doi: 10.1186/s12889-021-10648-5 (PMC8120725; doi:10.1186/s12889-021-10648-5)
Supplement: Supplementary file 1 — Additional file 1. Survey questions used to assess public awareness and practices related to antimicrobial resistance, China, 2019 (English version of survey questions). [file 12889_2021_10648_MOESM1_ESM.doc]

**Additional file 1 –** Survey questions used to assess public awareness and practices related to antimicrobial resistance, China, 2019.

Demographics –

1. Please record your sex
   1. Male
   2. Female
2. What age are you now?
   1. 16-24
   2. 25-44
   3. 45-64
   4. 65+
3. In what province do you reside?
   1. Drop-down option
4. Which of the locations below best describe where you live?
   1. Urban (densely populated city/town)
   2. Suburban (in a suburb of a city/town)
   3. Rural (outside of a city/town – such as in a village or farming area)
5. What is the highest degree of level of school you have completed?
   1. Less than 12^th^ grade
   2. High school graduate
   3. Some college or Associate degree
   4. Bachelor’s degree
   5. Master’s, professional, or doctoral degree

Main Questionnaire

*Use of antibiotics*

1. When was the last time you took antibiotics?
   1. In the last month
   2. In the last 2-6 months
   3. In the last 7-12 months
   4. More than a year ago
   5. Never (if never – go to question #4)
   6. Can’t remember
2. At this last time, where did you get the antibiotics?
   1. Medical store/pharmacy
   2. Friend/family member
   3. Internet
   4. Saved from a previous time
   5. Hospital
   6. Can’t remember
3. At this last time, did you get advice from a doctor, nurse, or pharmacist on how to take them?
   1. Yes
   2. No
   3. Can’t remember

*Knowledge about antibiotics*

1. After starting antibiotics, when do you think you should stop taking them?
   1. When you feel better
   2. When you have taken all antibiotics as directed
   3. Don’t know
2. Do you think that the following conditions be treated with antibiotics? For each, indicate ‘yes’, or ‘no’
   1. HIV/AIDS
   2. Diarrhea
   3. Cold or flu
   4. Urinary tract infection (UTI)
   5. Fever
   6. Malaria
   7. Measles
   8. Skin or wound infection
   9. Sore throat
   10. Body aches
   11. Headaches
3. Have you heard the term “Antibiotic Resistance”?
   1. Yes
   2. No (if no, go to question 8)
4. Where did you hear this term? (check all that apply)
   1. Doctor or nurse
   2. Pharmacist
   3. Family member or friend
   4. Media (TV, radio, social media)
   5. Other
   6. Can’t remember
5. Please indicate where you think the following statements are ‘true’ or ‘false’
   1. Antibiotic resistance occurs when your body becomes resistant to antibiotics and they no longer work well.
   2. Many infections are becoming increasingly resistant to treatment by antibiotics.
   3. If bacteria are resistant to antibiotics, it can be very difficult or impossible to treat the infections they cause.
   4. Antibiotic resistance is an issue that could affect me or my family.
   5. Antibiotic resistance is an issue in other countries but not here.
   6. Antibiotic resistance is only a problem for people who take antibiotics regularly.
   7. Bacteria which are resistant to antibiotics can be spread from person to person.
   8. Antibiotic-resistant infections could make medical procedures like surgery, organ transplants and cancer treatment much more dangerous.
6. On the scale shown, how much do you agree the following actions would help address antibiotic resistance?
   1. People should use antibiotics only when they are prescribed by a doctor or nurse – Agree Strongly [5], Agree Slightly [4], Neither agree or disagree [3], Disagree Slightly [2], Disagree Strongly [1]
   2. Farmers should give fewer antibiotics to food producing animals - Agree Strongly [5], Agree Slight [4], Neither agree or disagree [3], Disagree Slightly [2], Disagree Strongly [1]
   3. People should not keep antibiotics and use them later for other illnesses - Agree Strongly [5], Agree Slightly [4], Neither agree or disagree [3], Disagree Slightly [2], Disagree Strongly [1]
   4. Parents should make sure all of their children’s vaccinations are up-to-date - Agree Strongly [5], Agree Slightly [4], Neither agree or disagree [3], Disagree Slightly [2], Disagree Strongly [1]
   5. People should wash their hands regularly - Agree Strongly [5], Agree Slightly [4], Neither agree or disagree [3], Disagree Slightly [2], Disagree Strongly [1]
   6. Doctors should only prescribe antibiotics when they are needed - Agree Strongly [5], Agree Slightly [4], Neither agree or disagree [3], Disagree Slightly [2], Disagree Strongly [1]
   7. Governments should reward the development of new antibiotics - Agree Strongly [5], Agree Slightly [4], Neither agree or disagree [3], Disagree Slightly [2], Disagree Strongly [1]
   8. Pharmaceutical companies should develop new antibiotics - Agree Strongly [5], Agree Slightly [4], Neither agree or disagree [3], Disagree Slightly [2], Disagree Strongly [1]

*Use of antibiotics in agriculture*

1. Do you think that antibiotics are widely used in agriculture in China?
   1. Yes
   2. No
   3. Don’t know
