# Supplementary material for: Using Weibo and WeChat social media channels to assess public awareness and practices related to antimicrobial resistance, China, 2019
Source: BMC Public Health. 2021 May 14;21:921. doi: 10.1186/s12889-021-10648-5 (PMC8120725; doi:10.1186/s12889-021-10648-5)
Supplement: Supplementary file 2 — Additional file 2. Provincial Map of China, by Region, 2019 (Map highlighting location of provinces within each of the three main regions in China – Eastern, Central, and Western China). [file 12889_2021_10648_MOESM2_ESM.doc]

**Additional file 2 -** Provincial Map of China, by Region, 2019


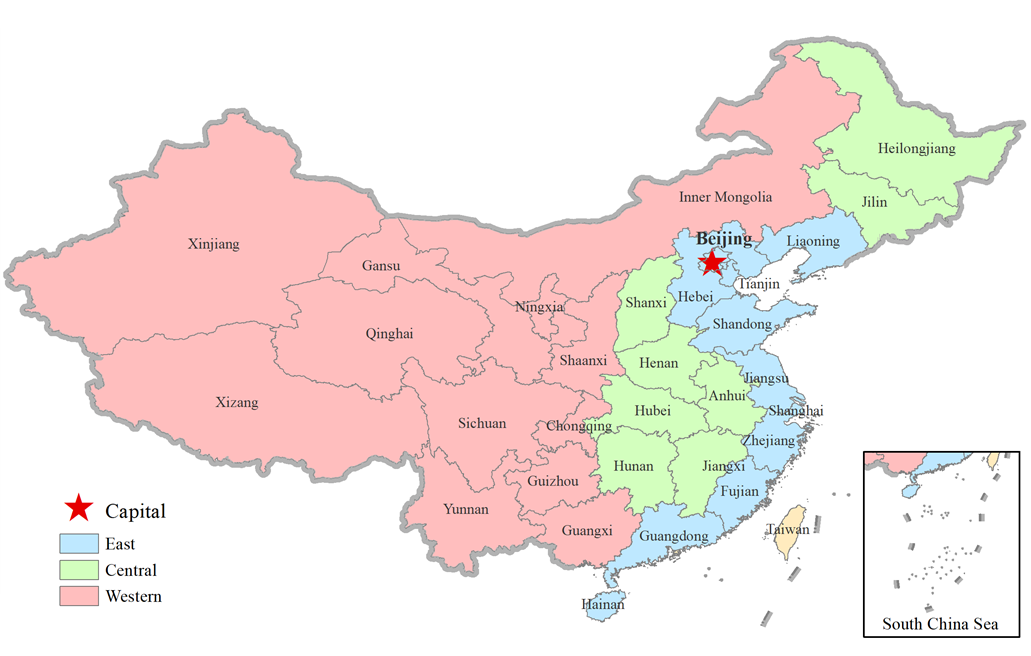


Created for this project using ArcGIS version 10.6 (ESRI, Redlands, California, USA). Provincial level boundary file obtained from the China National Bureau of Surveying and Mapping Geographic Information (former name).
