# Supplementary material for: Using Weibo and WeChat social media channels to assess public awareness and practices related to antimicrobial resistance, China, 2019
Source: BMC Public Health. 2021 May 14;21:921. doi: 10.1186/s12889-021-10648-5 (PMC8120725; doi:10.1186/s12889-021-10648-5)
Supplement: Supplementary file 3 — Additional file 3. Respondent feedback on approaches to help address antibiotic resistance from an online survey on antibiotic resistance, China, April 12–May 7, 2019 (Tables a, b). [file 12889_2021_10648_MOESM3_ESM.docx]

**Additional file 3.** Respondent feedback on approaches to help address antibiotic resistance, China, April 12 - May 7, 2019.

**Table A.** Respondents who answered “Agree Strongly” or “Agree Slightly” to “Do you think the following

actions would help address the problem of antibiotic resistance?” by urban/sub-urban place of residence.

|  | Urban  N (%) | Sub-urban  N (%) | Rural  N (%) |
| --- | --- | --- | --- |
| People should use antibiotics only when prescribed | 1,824 (87) | 432 (86) | 135 (80) |
| Farmers should give fewer antibiotics to animals | 1,883 (90) | 443 (87) | 140 (83) |
| People should not keep and use antibiotics later | 1,156 (55) | 291 (57) | 92 (55) |
| Parents should make sure children's vaccinations are up to date | 1,690 (81) | 426 (84) | 126 (75) |
| People should wash hands regularly | 2,061 (98) | 497 (98) | 165 (98) |
| Doctors should only prescribe antibiotics when needed | 2,028 (97) | 477 (94) | 157 (94) |
| Governments should reward the development of new antibiotics | 1,444 (69) | 340 (67) | 104 (62) |
| Pharmaceutical companies should develop new antibiotics | 1,397 (67) | 307 (61) | 87 (52) |

**Table B.** Respondents who answered “Agree Strongly” or “Agree Slightly” to “Do you think the following actions would help

address the problem of antibiotic resistance?” by education level.

|  | Less than  High School  N (%) | High School  Graduate  N (%) | Some Junior  College work  N (%) | Some Undergraduate work N (%) | At least some Graduate work  N (%) |
| --- | --- | --- | --- | --- | --- |
| People should use antibiotics only when prescribed | 126 (75) | 301 (85) | 594 (86) | 1,119 (88) | 251 (89) |
| Farmers should give fewer antibiotics to animals | 135 (81) | 318 (90) | 627 (90) | 1,136 (89) | 250 (89) |
| People should not keep & use antibiotics later | 95 (57) | 200 (57) | 397 (57) | 695 (54) | 152 (54) |
| Parents should make sure children's vaccinations are up to date | 137 (82) | 294 (83) | 564 (81) | 1,021 (80) | 226 (80) |
| People should wash hands regularly | 163 (98) | 347 (98) | 684 (99) | 1,254 (98) | 275 (98) |
| Doctors should only prescribe antibiotics when needed | 150 (90) | 336 (95) | 666 (96) | 1,236 (97) | 274 (98) |
| Governments should reward the development of new antibiotics | 115 (69) | 247 (70) | 456 (66) | 873 (68) | 197 (70) |
| Pharmaceutical companies should develop new antibiotics | 101 (61) | 221 (62) | 414 (60) | 860 (67) | 195 (69) |
